# Inadequate socialisation, inactivity, and urban living environment are associated with social fearfulness in pet dogs

**DOI:** 10.1038/s41598-020-60546-w

**Published:** 2020-02-26

**Authors:** Jenni Puurunen, Emma Hakanen, Milla K. Salonen, Salla Mikkola, Sini Sulkama, César Araujo, Hannes Lohi

**Affiliations:** 1 Department of Veterinary Biosciences and Department of Medical and Clinical Genetics, University of Helsinki, Helsinki, Finland; 2 Folkhälsan Research Center, Helsinki, Finland

**Keywords:** Animal behaviour, Behavioural ecology

## Abstract

Problematic behaviours are severe welfare issues for one of the world’s most popular pets, the domestic dog. One of the most prevalent behavioural problem that causes distress to dogs is social fearfulness, meaning fear of conspecifics or unfamiliar people. To identify demographic and environmental factors associated with fear of dogs and strangers, logistic regression was utilised with a large dataset of 6,000 pet dogs collected through an owner-filled behavioural survey. Social fearfulness was associated with several factors, including urban environment, poor socialisation during puppyhood, infrequent participation in training and other activities, small body size, female sex, and neutering. In addition, we identified several breed differences, suggesting a genetic contribution to social fearfulness. These findings highlight the role of inadequate socialisation, inactivity, and urban living environmental in fear-related behavioural problems in dogs. Improvements in the management and breeding practices of dogs could, therefore, enhance the welfare of man’s best friend.

## Introduction

Fear is a major welfare problem in pet dogs^1^. As a transient feeling aroused by specific stimuli, fear is a normal, fundamental emotion conserved among species which aids an individual to survive from threatening situation^2–4^. Fearfulness, on the other hand, is a personality trait^5^. If fear is excessive, prolonged, or generalised in nature, fearfulness becomes a behavioural problem which can interfere with the normal performance of the dog, causing high levels of distress or anxiety and increasing the risk of diseases or even decrease lifespan^1,6^. Moreover, problematic behaviours might have a negative valence not only on the wellbeing of the dog but also on the wellbeing of its owner^6–9^. Undesirable behaviours, such as excessive fearfulness, are the leading cause for relinquishment or even euthanasia of pet dogs worldwide^10–14^. In the worst case, fearful dogs may resort to aggression and cause public health threats^15–17^, highlighting the gravity of canine fearfulness.

Based on the stimulus eliciting fear, fearfulness can be divided into two separate categories in dogs: social and non-social fearfulness^5^. Social fearfulness encompasses fear of conspecifics or unfamiliar people, whereas non-social fearfulness includes fear of different stimuli, such as loud noises, novel situations, or heights and surfaces. Fearfulness is one of the most frequent canine behavioural problems as the prevalence of fearfulness ranges from 26.2% even to 44%^16–19^, and around 10–19% of dogs show fear of strangers or dogs^17,19,20^.

Many behavioural traits are complex. Multiple genes with only small effects^21^, a plethora of environmental factors^22^, and the complex interplay between these genetic and non-genetic factors^23^ all contribute to the development behaviour. Fearfulness in dogs is moderately heritable and associated with some candidate loci and genes^24–28^ in addition to environmental effects^22,29–33^. However, more research is needed to reveal the genetic risk variants and environmental factors associated with social fearfulness in dogs. Enhanced understanding of these genetic and environmental factors interacting together, leading to the onset of a behavioural problem, would give us tools to better recognise, manage, and prevent these conditions.

As a part of a larger population-based canine behavioural survey with over 13,700 participants, the aim of this study was to investigate the demographic and environmental factors associated with social fearfulness in Finnish pet dogs. By identifying the factors increasing the risk for social fear-related problems, we could improve the wellbeing of pet dogs.

## Results

### Study cohort and demographics

We studied the demographic and environmental factors associated with fear of dogs and fear of strangers in datasets of 5,973 and 5,932 dogs, respectively. In the ‘fear of dogs’ data, the numbers of non-fearful and fearful dogs were 4,806 and 1,167, respectively. The age of the dogs in this dataset varied from 2 months to 17 years (mean age 4.6 years). In the ‘fear of strangers’ data, the numbers of non-fearful and fearful dogs were 5,036 and 896, respectively. The age of the dogs in this dataset varied from 2 months to 17 years (mean age 4.7 years). 51% of the dogs were females in both datasets. More detailed demographics and the lists of included breeds and the number of individuals per breed are presented in the Supplementary Table S1.

### Demographic and environmental factors associated with fear of dogs

**Figure 1:**
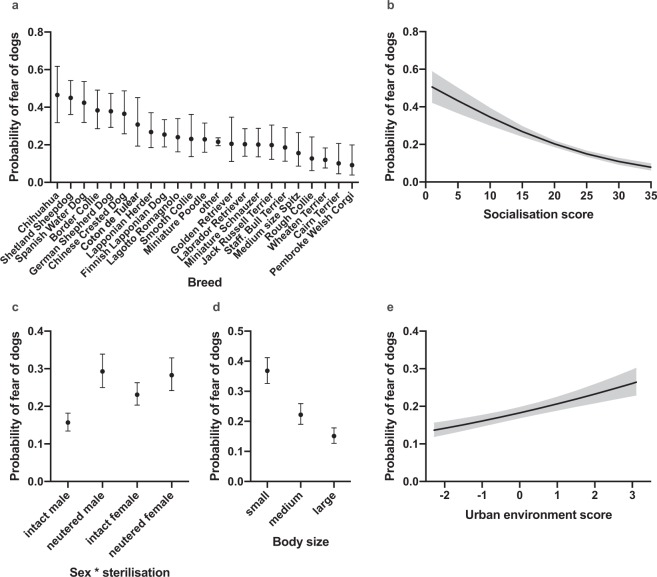
The effects of breed, socialisation, interaction between sex and sterilisation, body size, and living environment on fear of dogs in the logistic regression analysis. (**a**) Breeds differed in the likelihood of showing fear of dogs, with Chihuahua being the most and Pembroke Welsh Corgi the least fearful breed. (**b**) Dogs that had less socialisation experiences at the age of 7–16 weeks were more afraid of other dogs. (**c**) Intact males were less likely afraid of dogs than intact females, but no difference was observed between neutered males and females. In addition, intact individuals were less fearful in both sexes when compared to neutered individuals. (**d**) Small sized dogs were more likely fearful than medium and large sized dogs. There was also a difference between medium and large sized dogs. (**e**) Dogs living in a more urban environment had a higher likelihood of showing fear of dogs. Grey lines (**b**,**e**) and error bars (**a**,**c**,**d**) indicate 95% confidence limits. N = 5,973.

Logistic regression analysis identified several demographic and environmental factors associated with fear of dogs, including socialisation score, breed, body size, age, urban environment score, activities/training, daily exercise, and the interaction of sex and sterilisation (Table 1, Fig. 1).

**Table 1:** Associations between the demographic and environmental variables with fear of dogs and fear of strangers in the logistic regression analyses.

Variable	Fear of dogs			Fear of strangers		
	χ^2^	p-value	DF	χ^2^	p-value	DF
Age	8.40	0.004*	1	0.24	0.626*	1
Age^2	12.67	<0.001*	1	3.27	0.070*	1
Sex	31.75	<0.001*	1	4.86	0.027*	1
Sterilisation	48.65	**<0.001**	1	29.52	**<0.001**	1
Sex*Sterilisation	12.29	**0.003**	1			
Breed	128.30	**<0.001**	22	131.57	**<0.001**	19
Body size	115.96	**<0.001**	2	10.15	**0.027**	2
Socialisation score	82.91	<0.001*	1	129.71	<0.001*	1
Urban environment score	33.46	**<0.001**	1	14.52	**0.001**	1
Activities/training	29.20	**<0.001**	2	15.39	**0.003**	2
Daily exercise	13.36	**0.015**	3			
Family size				11.81	0.067	4
Weaning age				8.93	0.097	3

P-values are controlled for false discovery rate except for *a priori* contrasts. *A priori* effects are denoted with. *Significant effects are emboldened (p-value < 0.05). N = 5,973 (fear of dogs) and N = 5,932 (fear of strangers).

In the logistic regression model, socialisation score, body size, and breed had the strongest associations with fear of dogs. Dogs with less socialisation during puppyhood were more likely to show fear of dogs (χ^2^ = 82.91, DF = 1, p < 0.001) (Table 1, Fig. 1). Small dogs were more likely fearful when compared to both large (OR = 3.29, p < 0.001) and medium sized (OR = 2.04, p < 0.001) dogs, and there was also a significant difference between medium and large dogs (OR = 1.61, p < 0.001) (Supplementary Table S2, Fig. 1). We discovered behavioural differences between breeds. Chihuahua, Shetland Sheepdog, and Spanish Water Dog were the most fearful breeds whereas Pembroke Welsh Corgi, Cairn Terrier, and Wheaten Terrier were the least fearful breeds (Fig. 1). The largest pairwise differences were seen between Chihuahua and Pembroke Welsh Corgi (OR = 8.64, p < 0.001), Shetland Sheepdog and Pembroke Welsh Corgi (OR = 8.13, p < 0.001), and Spanish Water Dog and Pembroke Welsh Corgi (OR = 7.32, p < 0.001). Cairn Terrier also had a significantly lower likelihood of being fearful when compared to Chihuahua (OR = 0.13, p < 0.001), Shetland Sheepdog (OR = 0.14, p < 0.001), and Spanish Water Dog (OR = 0.15, p < 0.001). Significant pairwise breed differences are summarised in the Supplementary Table S3 and all pairwise breed differences are presented in the Supplementary Dataset. Based on previous research on breed differences of social fearfulness, we made an *a priori* hypothesis that Chihuahua, Jack Russell Terrier, Lagotto Romagnolo, and Shetland Sheepdog would be more fearful than German Shepherd Dog, Golden Retriever, Labrador Retriever, and Staff. Bull Terrier. Indeed, the first breed group was more likely afraid of dogs than the latter breed group (OR = 1.57, p = 0.024) (Supplementary Table S2).

There was an association between the age of the dog and fear of dogs, indicating that dogs from two to eight years old had the highest probability of being fearful, but this likelihood decreased after eight years of age (linear effect: χ^2^ = 8.40, DF = 1, p = 0.004; quadratic effect: χ^2^ = 12.67, DF = 1, p < 0.001) (Table 1, Supplementary Fig. S1). There was a significant interaction between sex and sterilisation of the dog, as intact males were less likely to show fear of dogs when compared to intact females (OR = 0.62, p < 0.001), but no difference was observed between neutered males and females (OR = 1.10, p = 0.807). Moreover, intact dogs were less likely fearful than neutered dogs in both sexes (intact male vs. neutered male: OR = 0.45, p < 0.001; intact female vs. neutered female: OR = 0.76, p = 0.036) (Supplementary Table S2, Fig. 1).

Dogs living in a more urban environment (χ^2^ = 33.46, DF = 1, p < 0.001) (Table 1, Fig. 1), participating less frequently in activities and training, and getting less daily exercise had higher probabilities of being afraid of dogs (Table 1, Supplementary Table S2, Supplementary Fig. S1). Dogs participating in activities only seldom or never were more likely fearful than dogs training sometimes (OR = 1.54, p < 0.001) or weekly (OR = 1.56, p < 0.001). Dogs getting less than one hour of exercise per day were more likely fearful than dogs exercising more than three hours daily (OR = 1.58, p = 0.022). Dogs exercising 1–2 hours per day were also more likely fearful when compared with dogs exercising more than three hours in a daily basis (OR = 1.50, p = 0.005).

### Demographic and environmental factors associated with fear of strangers

**Figure 2:**
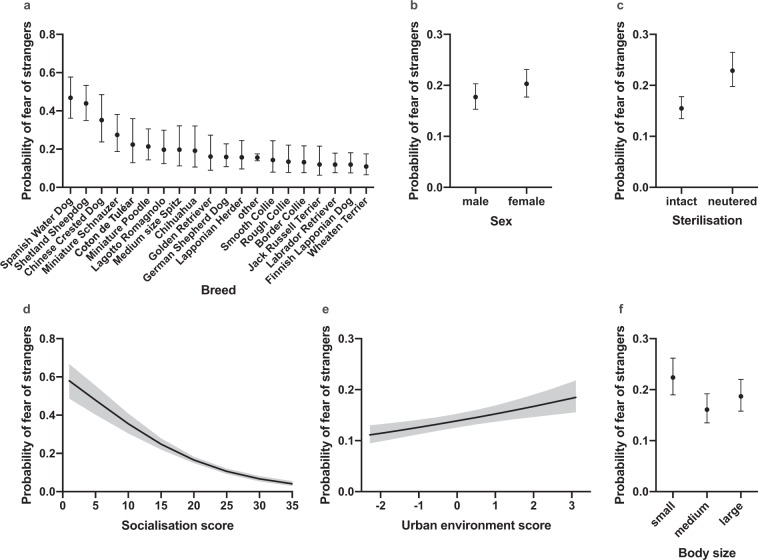
The effects of breed, sex, sterilisation, socialisation, living environment, and body size on fear of strangers in the logistic regression analysis. (**a**) Breeds differed in their likelihood of showing fear of strangers, with Spanish Water Dog being the most and Wheaten Terrier the least fearful breed. (**b**) Female dogs were more afraid of strangers than male dogs. **(c)** Intact dogs showed less fear of strangers than neutered dogs. (**d**) Dogs that had less socialisation experiences in the age of 7–16 weeks showed more fear of strangers. (**e**) Dogs living in a more urban environment had a higher likelihood of showing fear of strangers. (**f**) Small sized dogs were more afraid of strangers than medium sized dogs. Grey lines (**d**,**e**) and error bars (**a**,**b**,**c**,**f**) indicate 95% confidence limits. N = 5,932.

The best model explaining the difference between fearful and non-fearful dogs included several demographic and environmental factors, including socialisation score, breed, age, sex, sterilisation, body size, urban environment score, activities/training, weaning age, and family size (Table 1, Fig. 2).

Socialisation score and breed had the strongest associations with fear of strangers in the logistic regression analysis (Table 1, Fig. 2). Dogs with less socialisation experiences were more likely afraid of strangers (χ^2^ = 129.71, DF = 1, p < 0.001) (Table 1, Fig. 2). We discovered behavioural differences between breeds. Spanish Water Dog, Shetland Sheepdog, and Chinese Crested Dog were the most fearful breeds whereas Wheaten Terrier, Finnish Lapponian Dog, and Labrador Retriever were the least fearful breeds (Fig. 2). The largest pairwise differences were seen between Spanish Water Dog and Wheaten Terrier (OR = 7.20, p = 0.001), Spanish Water Dog and Finnish Lapponian Dog (OR = 6.53, p = 0.001), Spanish Water Dog and Labrador Retriever (OR = 6.52, p = 0.001), Spanish Water Dog and Jack Russell Terrier (OR = 6.43, p = 0.001), and Shetland Sheepdog and Wheaten Terrier (OR = 6.40, p = 0.001). Significant pairwise breed differences are summarised in the Supplementary Table S5 and all pairwise breed differences are presented in the Supplementary Dataset. Based on previous publications, we made an *a priori* hypothesis that Chihuahua, Jack Russell Terrier, Lagotto Romagnolo, and Shetland Sheepdog would be more fearful than German Shepherd Dog, Golden Retriever, and Labrador Retriever. We detected a significant difference between the groups, with the first breed group being more likely afraid of strangers than the latter one (OR = 1.65, p = 0.031) (Supplementary Table S4).

Dogs showing fear of strangers were more often females (OR = 0.85, p = 0.027) and neutered (OR = 0.62, p < 0.001) (Table 1, Supplementary Table S4, Fig. 2). Body size was also associated with fear of strangers, but the association was evident only between small and medium sized dogs (OR = 1.50, p = 0.01) (Supplementary Table S4, Fig. 2). Additionally, fearful dogs lived in more urban environments (χ^2^ = 14.54, DF = 1, p = 0.001) (Table 1, Fig. 2) and participated less frequently in activities and training. Dogs participating in activities only seldom or never were more likely fearful than dogs participating sometimes (OR = 1.36, p = 0.013) or weekly (OR = 1.44, p = 0.001) (Supplementary Fig. S2).

Age was not associated with fear of strangers (linear effect: χ^2^ = 0.24, DF = 1, p = 0.626; quadratic effect: χ^2^ = 3.27, DF = 1, p = 0.070), and the overall effects of family size and weaning age of the dog were not significant after controlling for false discovery rate (Table 1, Supplementary Fig. S2). However, when examining pairwise comparisons between dogs living in different sized families, we found that dogs living with one (‘single’) or two adults (‘couple’) were less likely to show fear of strangers that dogs living in families with two children (single vs. two children family: OR = 0.67, p = 0.015, couple vs. two children family: OR = 0.74, p = 0.046) (Supplementary Table S4, Supplementary Fig. S2). In addition, dogs weaned later than eight weeks of age had a higher likelihood to be afraid of strangers than dogs weaned at a normal age (from seven to eight weeks of age; OR = 0.80, p = 0.014) (Supplementary Table S4, Supplementary Fig. S2).

## Discussion

We investigated the demographic and environmental factors associated with two subtraits of canine social fearfulness, fear of dogs and fear of strangers, in a large behavioural survey data of 6,000 dogs using logistic regression. Social fearfulness was associated with several factors, which were almost identical in both subtraits. To sum up, fearful dogs were less socialised during puppyhood, small in body size, females and neutered, and participated less frequently in training and other activities. We also found a novel association between the living environment of the dog and social fearfulness, as dogs living in a more urban environment were more likely afraid of dogs and strangers. Moreover, we identified significant behavioural differences between dog breeds. These results reveal a comprehensive set of demographic and environmental factors associated with social fearfulness, but also highlight the role of genetic predisposition.

The level of socialisation during puppyhood (between seven weeks and four months of age) had the strongest association with both fear of dogs and fear of strangers. Fearful dogs had experienced less socialisation events in the age of 7–16 weeks than dogs showing no fear. Many mammals, including dogs, have a sensitive period for socialisation in the early postnatal life, during which the nervous system is immature and receptive for novel external stimuli^34,35^. Experiences and events taking place especially during 3–14 weeks of age can significantly affect dog’s behaviour throughout life^29,30,35^. In Finland, puppies are usually weaned and adopted to their new homes at the age of 7–8 weeks. This life change takes place during the sensitive period for socialisation, making the first weeks in the puppy’s new home crucial for the socialisation process.

Dogs weaned later than eight weeks of age were more likely to show fear of strangers than dogs weaned at a normal 7–8 weeks of age. This result might indicate that dogs weaned later encounter less socialisation experiences during the sensitive period than puppies moving to their new homes earlier. The breeder might not be able to provide an adequate amount of different kind of socialisation experiences when compared to the puppy’s new family. However, the association of inadequate socialisation and late weaning age might also reflect fearfulness already present at puppyhood. A fearful puppy might have to wait longer before it is adopted, and if it shows fear in the new home, it may not often meet unfamiliar people. Nevertheless, our results are consistent with earlier research, as it has been shown that early socialisation is necessary for normal social behaviour^22,29,35^ and that puppies weaned before the age of 12 weeks are bolder than puppies weaned later^33^. Therefore, development of social fearfulness may strongly depend on the diversity, amount, and proper timing of socialisation experiences.

We identified breed differences in fear of dogs and strangers. Spanish Water Dog and Shetland Sheepdog had a high probability of fear of dogs and strangers, whereas Wheaten Terrier had a low probability of both subtraits. Interestingly, the odds of showing fear greatly differed between the most and least fearful breeds, suggesting that some breeds might be more susceptible to fear than others. Many breed differences were similar in both fear of dogs and strangers, which could indicate comorbidity between these social fearfulness subtraits. However, some breeds only showed fear of dogs or strangers but not both. For example, Chihuahua and Border Collie rated high in fear of dogs but not in fear of strangers, whereas Miniature Schnauzer rated high in fear of strangers but not in fear of dogs.

Previous research has identified certain breeds that are more fearful than others^36–38^. These more fearful breeds include Chihuahua, Jack Russell Terrier, Lagotto Romagnolo, and Shetland Sheepdog. In contrast, German Shepherd Dog, Golden Retriever, Labrador Retriever, and Staff. Bull Terrier have been identified as less fearful breeds. We contrasted these fearful and non-fearful breeds and indeed discovered a difference in the probability of fear of dogs and strangers. However, more precise comparisons with previous research are difficult as the breed composition of study cohorts has been highly variable and moreover, instead of individual breeds, breed groups are often used to overcome small sample sizes in the statistical analyses. As a result, comparisons between findings of different studies might not be very meaningful.

We discovered that small dogs were more likely to show fear of dogs and strangers than large dogs. This result agrees with previous studies showing that fear of dogs and strangers decreases as the height of the dogs increases^39^, and that larger dogs are bolder in general^38,40,41^. Small sized dogs may feel more threatened by people and larger dogs due to the larger relative size difference and hence be more fearful. In addition, small dogs may not be trained and socialised enough^42^, as their small size makes them easier to handle even when they behave badly or unpredictably. Thus, undesirable behaviours may be more tolerated in small dogs than in larger conspecifics. For the same reason, behaviour may not be an important factor when making breeding plans, and thus, unwanted behaviours may accumulate. Another explanation is that body size could be genetically associated with reactivity and stress tolerance in dogs. However, as the most fearful breeds also include small sized breeds, despite having size as an explanatory variable in the models, the breed itself may be more important factor than the body size. This result emphasises the role of genetic predisposition in the development of behaviour and behavioural problems.

Interestingly, the living environment of the dog was associated with social fearfulness, as dogs living in an urban environment were more likely afraid of dogs and strangers than dogs living in more natural and agricultural areas. The relationship between behaviour and the living environment has not been studied in dogs before, but studies in humans have reported higher rates of mental disorders in urban areas when compared to rural environments^43–45^. However, the results have been somewhat inconsistent depending on, for example, the geographical location studied. In addition, the causality and mechanisms behind these associations are still mainly uncovered but studies have suggested several factors, such as diet, environmental toxins, stress, and social isolation^43,46^. As dogs share the environment with us, similar environmental factors could mediate this association in both species. In addition, urban areas, such as cities, may be very hectic and stressful environments to live as they are full of different stimuli, such as sudden and loud noises, potentially predisposing dogs to fear-related and other behavioural problems. On the other hand, dogs living in rural environments may meet other dogs and strangers less frequently due to the lower population density in rural areas. Nevertheless, in the lack of previous literature, no further conclusions can be made about the observed association between the urban environment and higher social fearfulness in dogs.

We found that dogs engaging in activities and training less frequently were more likely to show fear. In both subtraits, dogs participating only seldom in activities and training had a significantly higher likelihood to be fearful when compared to dogs participating in activities and training sometimes or weekly. Additionally, fear of conspecifics was associated with daily exercise. Dogs exercising less than two hours a day were more likely fearful than dogs getting more than three hours of exercise. Interestingly, lower levels of exercise have been previously associated with noise sensitivity and fear of strangers^22,47^. The observed associations may be explained by multiple different phenomena. Activities and exercise can fulfil dogs’ species-specific needs, acting as a way to reduce stress^48^. Therefore, dogs participating in activities more often may cope better in challenging situations. Previous research has demonstrated that both social and environmental enrichment together with exercise improves the wellbeing of dogs in kennels^49,50^. Moreover, dogs that engage more in activities and exercise may also habituate to the fearful stimuli. Additionally, dogs that are trained often likely interact more with their owners, possibly strengthening the human-dog bond^7,51^. Thus, the dog may feel safer in the presence of its owner and show less fear. On the other hand, owners may not be willing to train and exercise fearful dogs, as they may be difficult to handle and behave unpredictably, causing inconvenience and stress to the owner. Therefore, more research is needed to better understand the relationship between activities and social fearfulness.

Consistent sex differences in fearfulness were observed both in our dataset and in previous studies^22,33,36,37,52,53^. Females tend to be generally more fearful than males, and we observed the same trend in both fear of dogs and fear of strangers. In addition, neutered dogs were more likely afraid of dogs and strangers than intact dogs. Similar results have also been obtained earlier^22,52,53^. Moreover, we detected a significant interaction between sex and sterilisation in fear of dogs, as intact males were less fearful than intact females, but neutered males and females did not differ from each other. Interestingly, no interaction between sex and sterilisation was observed in fear of strangers. In some previous studies, neutering has been demonstrated as a risk factor for several problematic behaviours, potentially because of the hormonal changes caused by gonadectomy^54,55^. However, sometimes veterinarians may suggest neutering as a treatment for behavioural problems. In addition, dogs with undesirable behaviours are less likely used for breeding purposes, and thus they may be more likely neutered. Therefore, the causality of the relationship between neutering and social fearfulness remains elusive in our study.

Older dogs showed less fear of dogs, as the likelihood of fearfulness decreased after six years of age. A similar trend was also observed in fear of strangers, but the association was not significant. These results are supported by our previous studies: Tiira and Lohi (2015)^22^ discovered that younger dogs were generally more fearful, and Salonen *et al*.^17^ found that dogs 4–8 years old were most fearful of both dogs and strangers. However, opposite results have also been demonstrated^33,52^. The decline in fearfulness with age suggests that dogs may habituate to fearful stimuli and develop strategies to cope in these situations. Moreover, owners of fearful dogs may also adapt their actions, and learn to avoid situations in which the dog feels uncomfortable and shows fear.

In this survey study, we demonstrate that socialisation during puppyhood is strongly associated with social fearfulness in dogs, consistent with previous research. We have also replicated other findings from previous studies, as we show that fearful dogs are more often small, females and neutered, and participate less often in training and exercising. In addition, we identified several breed differences, suggesting that some breeds may be more vulnerable to develop social fear-related problems than others. Moreover, we report a novel association between the living environment of the dog and social fearfulness that requires further research. Most of the identified risk factors were common for both fear of dogs and fear of strangers, but we also identified some subtrait-specific risks, such as the association of daily exercise and fear of dogs, and the association of weaning age and fear of strangers. Our results indicate that social fearfulness in dogs is affected by multiple demographic and environmental factors and suggest that careful consideration and management of these factors could improve the wellbeing of pet dogs.

## Methods

### Data collection

An online owner-filled questionnaire was designed to collect information on canine behaviour in a pet dog population^17^. The questionnaire was divided into seven main sections based on different behavioural traits, including fear of dogs, strangers, and novel situations (labelled as ‘fear’); aggression toward human family members and strangers (labelled as ‘aggression’); fear of thunder, fireworks, and gunshot (labelled as ‘noise sensitivity’); fear of surfaces and heights; inattention and hyperactivity/impulsivity; separation anxiety; and compulsive behaviour. In addition, the questionnaire included several questions concerning the background and the current living environment of the dog, such as socialisation experiences during the age of 7–16 weeks, activities that the dog possibly participates in (e.g. dog shows, agility, or herding), amount of daily exercise, and the number of other dogs in the family. All the questions included in the behaviour questionnaire can be found as Supplementary material in the paper of Salonen *et al*.^17^.

In the questionnaire section concerning fear, the potential fearful reactions towards strangers and other dogs (labelled as ‘social fearfulness’) as well as novel situations (labelled as ‘non-social fearfulness’) were asked. As this paper focuses on studying the demographic and environmental factors associated with social fear in dogs, only the structure of the social fear subdivision is described in more detail here. If the owner answered that his/her dog reacts fearfully when meeting a stranger and/or a strange dog, we required him/her to report how often these reactions occur (rarely, 0–20% of occasions; sometimes, 20–40% of occasions; often, 40–60% of occasions; almost always, 60–100% of occasions; always, 100% of occasions). In addition, we asked him/her to more specifically indicate how the dog behaved in these situations (e.g. the dog withdraws when meeting a stranger). Moreover, if the owner responded that his/her dog does not react fearfully in those situations, he/she was asked to describe the dog’s behaviour more specifically to give us possibility to evaluate the behavioural reactions of the dog. The fear section of the questionnaire was previously shown to have high reliability and validity^56^.

As our aim was to reveal demographic and environmental factors associated with both fear of dogs and fear of strangers, two separate binomial (event/non-event) variables (‘fear of dogs’ and ‘fear of strangers’) describing these behaviours were derived from the questionnaire data. According to the owners, non-fearful dogs are reported to indicate no fear of other dogs (‘fear of dogs’) or strangers (‘fear of strangers’) whereas fearful dogs are reported to show fear of strange dogs (‘fear of dogs’) or strangers (‘fear of strangers’) in more than 40% of the occasions (Supplementary Table S6).

The questionnaire was advertised to dog owners on Facebook and via breed organisations. We obtained an informed consent from all participants and informed them that the questionnaire answers would be used for research. We emphasised that all data will remain confidential and that individual dogs and owners cannot be identified from the published results. Questionnaire replies were collected during 2015–2018.

### Demographic and environmental variables

Before statistical analyses, some demographic and environmental variables derived from the questionnaire were edited and some new variables were created. First, only breeds with enough individuals in both non-fearful and fearful groups (>10 individuals/group) were selected for the analyses. Individuals in other breeds were grouped together under ‘other’ breed group. The selected breeds included Border Collie, Cairn Terrier, Chihuahua, Chinese Crested Dog, Coton de Tuléar, Finnish Lapponian Dog, German Shepherd Dog, Golden Retriever, Irish Soft Coated Wheaten Terrier (labelled as ‘Wheaten Terrier’), Jack Russell Terrier, Labrador Retriever, Lagotto Romagnolo, Lapponian Herder, Medium size Spitz, Miniature Poodle (including Toy, Miniature, and Medium Poodle), Miniature Schnauzer, Pembroke Welsh Corgi, Rough Collie, Shetland Sheepdog, Smooth Collie, Spanish Water Dog, and Staffordshire Bull Terrier (labelled as ‘Staff. Bull Terrier’) (Supplementary Table S1). Mixed breed dogs were also included in the data. In the ‘fear of strangers’ analysis, Cairn Terrier, Pembroke Welsh Corgi, and Staff. Bull Terrier were included in the group ‘other’ as they had too few individuals for the logistic regression analysis.

Second, we created a new categorical variable, body size, based on the average heights of breeds. The heights were drawn from FCI and AKC standards, when available. For other breeds, with no FCI or AKC standards, the average heights were determined based on heights reported by breed clubs. If different heights were reported for females and males, the mean was calculated and used as the average height. Individuals with breed average heights of ≤35 cm, 36–49 cm, or ≥50 cm belonged to small, medium, or large categories, respectively. When body size was included in the analyses, mixed breed dogs were excluded as their body size could not be determined.

Third, a continuous variable labelled as socialisation score was calculated based on the frequency of socialisation events when the dog was 7–16 weeks old. The score is a sum of the frequencies (0 = never; 1 = 1–2 times during the puppyhood; 2 = 1–2 times during the puppyhood to 2 times per month; 3 = twice a month to twice a week; 4 = twice a week to once a day; 5 = several times a day) the dog met unfamiliar men, women and children, unfamiliar adult dogs, visited city or other place with traffic and many people, and travelled by car or by bus.

Fourth, a continuous variable labelled as urban environment score describing the environmental land-use around the dog’s home was created. We derived the geographical coordinates for each home from addresses provided by the dog owners, and calculated the coverage of three land-use types, artificial surfaces, agricultural areas, and forests and semi-natural areas within a three-kilometre range around the homes using the land-use database CORINE2012. Finally, we simplified the coverages into one continuous variable with principal component analysis (PCA). The higher the urban environment score, the more urban was the environment.

Variables derived from the questionnaire data and included in the analyses are described in more detail in the Supplementary Table S6.

### Statistical analyses

Logistic regression was used to find demographic and environmental factors associated with two subtraits of social fearfulness, fear of dogs and fear of strangers. Initially, the data consisted of 13,715 dogs in 264 breeds. After including only fearful and non-fearful dogs and excluding individuals with missing or incomplete responses, the data consisted of 5,343 dogs in ‘fear of dogs’ and 5,858 in ‘fear of strangers’. The subtraits were used as binary response variables in the analyses: fearful and non-fearful dogs constituted the event and the non-event, respectively. Based on previous literature, we included several explanatory variables in the analyses. Demographic explanatory variables included age, sex, sterilisation, breed, and body size. Environmental explanatory variables included socialisation score, weaning age, urban environment score, daily exercise, activities/training, owner’s dog experience, dogs in the family, family size, and daily time spent alone. To find the models with the best fit, a forward stepwise model selection by Akaike Information Criterion (AIC) values was used and was initiated with a starter model of sex and age in both ‘fear of dogs’ and ‘fear of strangers’ analyses. The AIC model selection process and the final models are presented in the Supplementary Table S7. In the ‘fear of dogs’ model, the interaction between sex and sterilisation was significant and improved the AIC value of the model. Therefore, the interaction term (sex*sterilisation) was included in the final model. To maximise the sample sizes, new subsets of the initial data were created to include only those variables included in the final models. This resulted in datasets of 5,973 and 5,932 individuals for ‘fear of dogs’ and ‘fear of strangers’, respectively.

After model selection, generalised additive models were fitted with the package ‘gam’^57^ in R to test the linearity assumption of continuous variables. If the linearity assumption of a variable was not met, the variable squared was also included in the model (e.g. age^2). Packages ‘broom’^58^ and ‘dplyr’^59^ in R were used to inspect possible outliers in the datasets. Standardised residuals were plotted using the package ‘ggplot2’^60^ in R. Generalised variance inflation factor (gVIF) was utilised to test multicollinearity with the package ‘car’^61^, and the area under the receiver operator characteristic curve (AUC) was calculated to estimate how well the model predicted the event and the non-event using the package ‘pROC’^62^ in R. The predictive abilities of both models were reasonable (AUC(fear of dogs) = 0.728 and AUC(fear of strangers) = 0.688).

Estimated marginal means were calculated for categorical explanatory variables with the package ‘emmeans’^63^ in R. The effects of continuous explanatory variables (adjusting for other variables in the models) were obtained with the package ‘effects’^64^ in R. To obtain the overall effects of the explanatory variables, analysis of variance (ANOVA) was conducted with the package ‘car’^61^ in R. Based on previous literature, we determined several contrasts between levels of explanatory variables *a priori*. First, we requested a contrast between potentially more fearful (Chihuahua, Jack Russell Terrier, Lagotto Romagnolo, and Shetland Sheepdog) and less fearful (German Shepherd Dog, Golden Retriever, Labrador Retriever, and Staff. Bull Terrier) breeds^36–38^. Second, we hypothesised that female dogs would be more fearful than male dogs^33,36,37,52,53^. Third, we hypothesised that large dogs would differ from small dogs in their behaviour^38–41^. Fourth, contrast between early weaning (weaned <7 weeks of age) and normal weaning age (7–8 weeks of age) as well as contrast between normal weaning age and late weaning age (>8 weeks of age) were requested^22,33^. Fifth, we hypothesised that younger dogs would be more fearful^22^. Finally, we made a hypothesis that dogs with less socialisation (i.e. lower socialisation scores) would show more fearful behaviour^22^. The hypotheses and contrasts were the same in both ‘fear of dogs’ and ‘fear of strangers’ analyses, except for weaning age which was only included in the ‘fear of strangers’ model. Furthermore, in the ‘fear of strangers’ analysis, the breed contrast was as follows: Chihuahua, Jack Russell Terrier, Lagotto Romagnolo, and Shetland Sheepdog versus German Shepherd Dog, Golden Retriever, and Labrador Retriever (Staff. Bull Terrier was left out due to a low sample size).

*A priori* contrasts and all pairwise comparisons between levels of the included categorical variables were examined with the package ‘emmeans’^63^ in R. As we had several categorical variables, the number of pairwise comparisons was high and therefore all obtained p-values, except contrasts chosen *a priori*, were controlled for false discovery rate (FRD). The significance cut-off was set at p-value <0.05.

All statistical analyses were conducted in R version 3.6.1^65^.

## Supplementary information

Supplementary information.

Supplementary information 2.

## Data Availability

The anonymised data used in this study can be found as Supplementary material in the paper of Salonen *et al*.^17^.
