# Supplementary material for: Inadequate socialisation, inactivity, and urban living environment are associated with social fearfulness in pet dogs
Source: Sci Rep. 2020 Feb 26;10:3527. doi: 10.1038/s41598-020-60546-w (PMC7044223; doi:10.1038/s41598-020-60546-w)
Supplement: Supplementary file 2 — Supplementary information 2. [file 41598_2020_60546_MOESM2_ESM.pdf]

Supplementary Information

Inadequate socialisation, inactivity, and urban living environment are associated with social fearfulness in pet dogs

Jenni Puurunen[1,2], Emma Hakanen[1,2], Milla K. Salonen[1,2], Salla Mikkola[1,2], Sini Sulkama[1,2], César Araujo[1,2], Hannes Lohi[1,2*]

[1] Department of Veterinary Biosciences and Department of Medical and Clinical Genetics, University of Helsinki, Helsinki, Finland

[2] Folkhälsan Research Center, Helsinki, Finland

* Corresponding author:

Hannes Lohi, PhD, Professor

Email: hannes.lohi@helsinki.fi (HL)

Supplementary Table S1. Descriptive statistics. SD = standard deviation. N(fear of dogs) = 5,973, N(fear of strangers) = 5,932.

Fear of dogs

		mean	SD
Age (years)		4.61	3.26
Socialisation score		21.85	4.34
Urban environment score		-0.038	1.39
		**N**	**%**
Sex	Female	3071	51.41
	Male	2902	48.59
Sterilisation	Intact	4409	73.82
	Neutered	1564	26.18
Breed	Border Collie	156	2.61
	Cairn Terrier	42	0.70
	Chihuahua	50	0.84
	Chinese Crested Dog	72	1.21
	Coton de Tuléar	47	0.79
	Finnish Lapponian Dog	213	3.57
	German Shepherd Dog	204	3.42
	Golden Retriever	81	1.36
	Jack Russell Terrier	65	1.09
	Labrador Retriever	221	3.7
	Lagotto Romagnolo	124	2.08
	Lapponian Herder	121	2.03
	Medium size Spitz	52	0.87
	Miniature poodle	121	2.03
	Miniature Schnauzer	99	1.66
	Other	3491	58.45
	Pembroke Welsh Corgi	37	0.62
	Rough Collie	126	2.11
	Shetland Sheepdog	165	2.76
	Smooth Collie	99	1.66
	Spanish Water Dog	101	1.69
	Staff. Bull Terrier	90	1.51
	Wheaten Terrier	196	3.28
Body size	Small	1340	22.43
	Medium	2004	33.55
	Large	2629	44.01
Activities/training	Never/seldom	1386	23.20
	Sometimes	1714	28.70
	At least weekly	2873	48.10
Daily exercise	< 1 hour	389	6.51
	1-2 hours	2358	39.48
	2-3 hours	2354	39.41
	> 3 hours	872	14.60

Fear of strangers

		mean	SD
Age (years)		4.65	3.28
Socialisation score		22.02	4.19
Urban environment score		0.008	1.39
		**N**	**%**
Sex	Female	3023	50.96
	Male	2909	49.04
Sterilisation	Intact	4346	73.26
	Neutered	1586	26.74
Breed	Border Collie	142	2.39
	Chihuahua	49	0.83
	Chinese Crested Dog	63	1.06
	Coton de Tuléar	52	0.88
	Finnish Lapponian Dog	242	4.08
	German Shepherd Dog	210	3.54
	Golden Retriever	80	1.35
	Jack Russell Terrier	75	1.26
	Labrador Retriever	217	3.66
	Lagotto Romagnolo	117	1.97
	Lapponian Herder	135	2.28
	Medium size Spitz	60	1.01
	Miniature poodle	122	2.06
	Miniature Schnauzer	96	1.62
	Other	3589	60.50
	Rough Collie	125	2.11
	Shetland Sheepdog	168	2.83
	Smooth Collie	93	1.57
	Spanish Water Dog	108	1.82
	Wheaten Terrier	189	3.19
Body size	Small	1371	23.11
	Medium	2061	34.74
	Large	2500	42.14
Activities/training	Never/seldom	1402	23.63
	Sometimes	1700	28.66
	At least weekly	2830	47.71
Family size	Single	1166	19.66
	Couple	2632	44.37
	One child	699	11.78
	Two children	731	12.32
	Larger family	704	11.87
Weaning age	< 7 weeks	526	8.87
	at 7 weeks	1934	32.60
	at 8 weeks	2167	36.53
	> 8 weeks	1305	22.00

Supplementary Table S2. Contrasts between levels of categorical variables in the 'fear of dogs' logistic regression analysis. P-values are controlled for false discovery rate except for *a priori* contrasts. *A priori* effects are denoted with *. Significant effects are emboldened (p-value < 0.05). OR = odds ratio, CI = confidence limit. N = 5,973.

**Variable**	**Contrast**	**OR**	**Lower 95%CI**	**Upper 95%CI**	**p**
Sex	Male vs. female	0.65	0.480	0.867	**0.0037***
Sterilisation	Intact vs. neutered	0.34	0.248	0.471	**< 0.001**
Sex*sterilisation	Intact male vs. neutered male	0.45	0.358	0.562	**< 0.001**
	Intact female vs. neutered female	0.76	0.616	0.941	**0.036**
	Intact male vs. intact female	0.62	0.521	0.730	**< 0.001**
	Neutered male vs. neutered female	1.10	0.820	1.334	0.807
	Intact male vs. neutered female	0.47	0.377	0.584	**< 0.001**
	Intact female vs. neutered male	0.73	0.585	0.905	**0.017**
Body size	Small vs. medium	2.04	1.630	2.550	**< 0.001***
	Small vs. large	3.29	2.640	4.090	**< 0.001**
	Medium vs. large	1.61	1.280	2.030	**< 0.001**
Activities/training	Never/seldom vs. sometimes	1.54	1.278	1.850	**< 0.001**
	Never/seldom vs. at least weekly	1.56	1.305	1.860	**< 0.001**
	Sometimes vs. at least weekly	1.01	0.853	1.210	0.919
Daily exercise	< 1 hour vs. 1-2 hours	1.05	0.809	1.370	0.794
	< 1 hour vs. 2-3 hours	1.23	0.940	1.620	0.226
	< 1 hour vs. > 3hours	1.58	1.137	2.190	**0.022**
	1-2 hours vs. 2-3 hours	1.17	1.004	1.370	0.099
	1-2 hours vs. > 3 hours	1.50	1.178	1.900	**0.005**
	2-3 hours vs. > 3 hours	1.28	1.002	1.630	0.106
Breed	Chihuahua, Jack Russell Terrier, Lagotto Romagnolo, and Shetland Sheepdog vs. German Shepherd Dog, Golden Retriever, Labrador Retriever, and Staff. Bull Terrier.	1.57	1.060	2.330	**0.024***

Supplementary Table S3. Significant pairwise breed differences in the ‘fear of dogs’ analysis. All p-values are FDR-controlled. OR = odds ratio, CI = confidence limit. N = 5,973.

Breed contrast	OR	Lower 95%CI	Upper 95%CI	p
Border Collie vs. Cairn Terrier	5.54	2.137	14.340	0.003
Border Collie vs. Jack Russell Terrier	2.51	1.217	5.176	0.038
Border Collie vs. Labrador Retriever	2.44	1.351	4.402	0.013
Border Collie vs. Medium size Spitz	3.37	1.514	7.505	0.013
Border Collie vs. Miniature Schnauzer	2.47	1.291	4.707	0.022
Border Collie vs. Other	2.26	1.460	3.508	0.002
Border Collie vs. Pembroke Welsh Corgi	6.18	2.257	16.907	0.003
Border Collie vs. Rough Collie	4.27	1.783	10.204	0.006
Border Collie vs. Staff. Bull Terrier	2.73	1.305	5.710	0.025
Border Collie vs. Wheaten Terrier	4.56	2.335	8.892	< 0.001
Cairn Terrier vs. Chihuahua	0.13	0.046	0.361	< 0.001
Cairn Terrier vs. Chinese Crested Dog	0.20	0.075	0.509	0.005
Cairn Terrier vs. Coton de Tuléar	0.25	0.091	0.703	0.027
Cairn Terrier vs. German Shepherd Dog	0.18	0.073	0.467	0.003
Cairn Terrier vs. Lapponian Herder	0.31	0.116	0.808	0.047
Cairn Terrier vs. Shetland Sheepdog	0.14	0.055	0.345	< 0.001
Cairn Terrier vs. Spanish Water Dog	0.15	0.059	0.398	< 0.001
Chihuahua vs. Finnish Lapponian Dog	2.54	1.220	5.303	0.038
Chihuahua vs. Golden Retriever	3.37	1.297	8.755	0.038
Chihuahua vs. Jack Russell Terrier	3.51	1.543	7.993	0.013
Chihuahua vs. Labrador Retriever	3.41	1.578	7.369	0.009
Chihuahua vs. Lagotto Romagnolo	2.75	1.244	6.078	0.038
Chihuahua vs. Medium size Spitz	4.71	1.937	11.475	0.004
Chihuahua vs. Miniature Poodle	2.92	1.387	6.156	0.018
Chihuahua vs. Miniature Schnauzer	3.45	1.618	7.344	0.007
Chihuahua vs. Other	3.17	1.696	5.907	0.002
Chihuahua vs. Pembroke Welsh Corgi	8.64	2.935	25.423	< 0.001
Chihuahua vs. Rough Collie	5.96	2.189	16.250	0.003
Chihuahua vs. Staff. Bull Terrier	3.82	1.615	9.018	0.011
Chihuahua vs. Wheaten Terrier	6.37	2.858	14.206	< 0.001
Chinese Crested Dog vs. Medium size Spitz	3.12	1.387	7.020	0.021
Chinese Crested Dog vs. Other	2.10	1.265	3.469	0.016
Chinese Crested Dog vs. Pembroke Welsh Corgi	5.72	2.071	15.794	0.005
Chinese Crested Dog vs. Wheaten Terrier	4.22	2.072	8.588	< 0.001
Coton de Tuléar vs. Pembroke Welsh Corgi	4.41	1.506	12.932	0.023
Coton de Tuléar vs. Wheaten Terrier	3.26	1.471	7.204	0.015
Finnish Lapponian Dog vs. Pembroke Welsh Corgi	3.40	1.267	9.108	0.043
Finnish Lapponian Dog vs. Wheaten Terrier	2.51	1.390	4.515	0.010
German Shepherd Dog vs. Miniature Poodle	2.05	1.138	3.679	0.047
German Shepherd Dog vs. Pembroke Welsh Corgi	6.05	2.256	16.223	0.002
German Shepherd Dog vs. Staff. Bull Terrier	2.67	1.316	5.430	0.022
German Shepherd Dog vs. Wheaten Terrier	4.46	2.364	8.424	< 0.001
Golden Retriever vs. Shetland Sheepdog	0.32	0.140	0.709	0.019
Jack Russell Terrier vs. German Shepherd Dog	0.41	0.203	0.813	0.035
Jack Russell Terrier vs. Shetland Sheepdog	0.30	0.153	0.599	0.004
Labrador Retriever vs. German Shepherd Dog	0.42	0.240	0.729	0.010
Labrador Retriever vs. Shetland Sheepdog	0.31	0.174	0.558	< 0.001
Lagotto Romagnolo vs. Shetland Sheepdog	0.39	0.220	0.681	0.005
Lagotto Romagnolo vs. Wheaten Terrier	2.32	1.197	4.485	0.038

Lapponian Herder vs. Pembroke Welsh Corgi	3.64	1.306	10.145	0.039
Lapponian Herder vs. Shetland Sheepdog	0.45	0.257	0.780	0.017
Lapponian Herder vs. Wheaten Terrier	2.69	1.400	5.149	0.013
Medium size Spitz vs. German Shepherd Dog	0.30	0.140	0.656	0.011
Medium size Spitz vs. Shetland Sheepdog	0.23	0.105	0.483	< 0.001
Miniature Schnauzer vs. Chinese Crested Dog	0.44	0.227	0.848	0.041
Miniature Schnauzer vs. German Shepherd Dog	0.41	0.224	0.765	0.018
Miniature Schnauzer vs. Shetland Sheepdog	0.31	0.169	0.562	< 0.001
Other vs. German Shepherd Dog	0.45	0.306	0.665	< 0.001
Other vs. Shetland Sheepdog	0.34	0.232	0.486	< 0.001
Other vs. Wheaten Terrier	2.01	1.218	3.328	0.022
Rough Collie vs. Chinese Crested Dog	0.25	0.100	0.644	0.015
Rough Collie vs. German Shepherd Dog	0.24	0.103	0.559	0.005
Rough Collie vs. Shetland Sheepdog	0.18	0.075	0.424	< 0.001
Rough Collie vs. Spanish Water Dog	0.20	0.080	0.491	0.003
Shetland Sheepdog vs. Finnish Lapponian Dog	2.39	1.481	3.868	0.003
Shetland Sheepdog vs. Miniature Poodle	2.75	1.577	4.793	0.003
Shetland Sheepdog vs. Pembroke Welsh Corgi	8.13	3.057	21.618	< 0.001
Shetland Sheepdog vs. Staff. Bull Terrier	3.59	1.861	6.931	< 0.001
Shetland Sheepdog vs. Wheaten Terrier	6.00	3.366	10.679	< 0.001
Smooth Collie vs. Shetland Sheepdog	0.37	0.176	0.766	0.025
Spanish Water Dog vs. Finnish Lapponian Dog	2.16	1.243	3.735	0.022
Spanish Water Dog vs. Golden Retriever	2.86	1.218	6.695	0.045
Spanish Water Dog vs. Jack Russell Terrier	2.98	1.430	6.193	0.015
Spanish Water Dog vs. Labrador Retriever	2.89	1.521	5.490	0.006
Spanish Water Dog vs. Lagotto Romagnolo	2.33	1.246	4.354	0.026
Spanish Water Dog vs. Medium size Spitz	3.99	1.779	8.969	0.005
Spanish Water Dog vs. Miniature Poodle	2.48	1.338	4.582	0.015
Spanish Water Dog vs. Miniature Schnauzer	2.92	1.513	5.636	0.007
Spanish Water Dog vs. Other	2.68	1.699	4.231	< 0.001
Spanish Water Dog vs. Pembroke Welsh Corgi	7.32	2.655	20.176	< 0.001
Spanish Water Dog vs. Staff. Bull Terrier	3.23	1.591	6.572	0.006
Spanish Water Dog vs. Wheaten Terrier	5.40	2.858	10.197	< 0.001

Supplementary Table S4. Contrasts between levels of categorical variables in the ‘fear of strangers’ logistic regression analysis. P-values are controlled for false discovery rate except for *a priori* contrasts. *A priori* effects are denoted with *. Significant effects are emboldened (p-value < 0.05). OR = odds ratio, CI = confidence limit. N = 5,932.

**Variable**	**Contrast**	**OR**	**Lower 95%CI**	**Upper 95%CI**	**p**
Sex	Male vs. female	0.85	0.728	0.981	**0.027***
Sterilisation	Intact vs. neutered	0.62	0.517	0.733	**0.001**
Body size	Small vs. medium	1.50	1.167	1.930	**0.010**
	Small vs. large	1.25	0.996	1.570	0.055*
	Medium vs. large	0.83	0.656	1.060	0.322
Activities/training	Never/seldom vs. sometimes	1.36	1.115	1.660	**0.013**
	Never/seldom vs. at least weekly	1.44	1.188	1.740	**0.001**
	Sometimes vs. at least weekly	1.05	0.877	1.270	0.746
Family size	Single vs. couple	0.90	0.727	1.104	0.508
	Single vs. one child	0.79	0.594	1.038	0.236
	Single vs. two children	0.67	0.508	0.871	**0.015**
	Single vs. larger family	0.74	0.559	0.967	0.091
	Couple vs. one child	0.88	0.688	0.118	0.504
	Couple vs. two children	0.74	0.590	0.936	**0.046**
	Couple vs. larger family	0.82	0.648	1.040	0.261
	One child vs. two children	0.85	0.632	1.136	0.492
	One child vs. larger family	0.94	0.695	1.261	0.801
	Two children vs. larger family	1.11	0.828	1.473	0.687
Weaning age	<7 weeks vs. at 7 weeks	0.88	0.651	1.195	0.610
	<7 weeks vs. at 8 weeks	0.82	0.606	1.100	0.388
	<7 weeks vs. > 8 weeks	0.68	0.500	0.930	0.058
	At 7 weeks vs. at 8 weeks	0.93	0.770	1.113	0.606
	At 7 weeks vs. > 8 weeks	0.77	0.632	0.947	**0.048**
	At 8 weeks vs. > 8 weeks	0.84	0.690	1.012	0.179
	<7 weeks vs. 7-8 weeks	0.85	0.637	1.130	0.262
	7-8 weeks vs. >8 weeks	0.80	0.676	0.957	**0.014**
Breed	Chihuahua, Jack Russell Terrier, Lagotto Romagnolo, and Shetland Sheepdog vs. German Shepherd Dog, Golden Retriever, and Labrador Retriever.	1.65	1.050	2.600	**0.031***

Supplementary Table S5. Significant pairwise breed differences in the ‘fear of strangers’ analysis. All p-values are FDR-controlled. OR = odds ratio, CI = confidence limit. N = 5,932.

**Breed contrast**	**OR**	**Lower 95%CI**	**Upper 95%CI**	**p**
Border Collie vs. Chinese Crested Dog	0.28	0.124	0.635	0.013
Border Collie vs. Shetland Sheepdog	0.19	0.096	0.394	0.001
Border Collie vs. Spanish Water Dog	0.17	0.082	0.364	0.001
Chihuahua vs. Shetland Sheepdog	0.30	0.138	0.664	0.014
Chihuahua vs. Spanish Water Dog	0.27	0.118	0.613	0.010
Chinese Crested Dog vs. Finnish Lapponian Dog	4.03	1.909	8.498	0.002
Chinese Crested Dog vs. German Shepherd Dog	2.87	1.411	5.824	0.016
Chinese Crested Dog vs. Labrador Retriever	4.02	1.934	8.352	0.001
Chinese Crested Dog vs. Lapponian Herder	2.92	1.325	6.428	0.032
Chinese Crested Dog vs. Other	2.93	1.678	5.105	0.001
Chinese Crested Dog vs. Wheaten Terrier	4.44	2.024	9.722	0.001
Coton de Tuléar vs. Shetland Sheepdog	0.37	0.172	0.794	0.042
Coton de Tuléar vs. Spanish Water Dog	0.33	0.147	0.733	0.028
German Shepherd Dog vs. Shetland Sheepdog	0.24	0.136	0.431	0.001
Golden Retriever vs. Shetland Sheepdog	0.25	0.115	0.529	0.002
Jack Russell Terrier vs. Chinese Crested Dog	0.25	0.106	0.599	0.010
Jack Russell Terrier vs. Shetland Sheepdog	0.18	0.079	0.389	0.001
Labrador Retriever vs. Shetland Sheepdog	0.17	0.094	0.316	0.001
Lagotto Romagnolo vs. Shetland Sheepdog	0.31	0.169	0.582	0.001
Lapponian Herder vs. Shetland Sheepdog	0.24	0.127	0.443	0.001
Medium size Spitz vs. Shetland Sheepdog	0.31	0.147	0.670	0.014
Miniature Schnauzer vs. Finnish Lapponian Dog	2.80	1.380	5.675	0.019
Miniature Schnauzer vs. Labrador Retriever	2.79	1.403	5.558	0.016
Miniature Schnauzer vs. Other	2.03	1.234	3.351	0.024
Miniature Schnauzer vs. Wheaten Terrier	3.08	1.461	6.499	0.015
Other vs. Shetland Sheepdog	0.24	0.162	0.346	0.001
Rough Collie vs. Chinese Crested Dog	0.29	0.126	0.648	0.014
Rough Collie vs. Shetland Sheepdog	0.20	0.097	0.403	0.001
Rough Collie vs. Spanish Water Dog	0.18	0.083	0.373	0.001
Shetland Sheepdog vs. Finnish Lapponian Dog	5.81	3.291	10.263	0.001
Shetland Sheepdog vs. Miniature Poodle	2.88	1.595	5.192	0.003
Shetland Sheepdog vs. Wheaten Terrier	6.40	3.449	11.876	0.001
Smooth Collie vs. Chinese Crested Dog	0.31	0.131	0.729	0.030
Smooth Collie vs. Shetland Sheepdog	0.21	0.100	0.455	0.001
Smooth Collie vs. Spanish Water Dog	0.19	0.086	0.420	0.001
Spanish Water Dog vs. Finnish Lapponian Dog	6.53	3.519	12.131	0.001
Spanish Water Dog vs. German Shepherd Dog	4.65	2.486	8.698	0.001
Spanish Water Dog vs. Golden Retriever	4.57	2.053	10.161	0.001
Spanish Water Dog vs. Jack Russell Terrier	6.43	2.793	14.790	0.001
Spanish Water Dog vs. Labrador Retriever	6.52	3.395	12.519	0.001
Spanish Water Dog vs. Lagotto Romagnolo	3.58	1.849	6.948	0.001
Spanish Water Dog vs. Lapponian Herder	4.73	2.426	9.239	0.001
Spanish Water Dog vs. Medium size Spitz	3.59	1.615	7.967	0.010
Spanish Water Dog vs. Miniature Poodle	3.24	1.713	6.111	0.002
Spanish Water Dog vs. Miniature Schnauzer	2.34	1.196	4.556	0.048
Spanish Water Dog vs. Other	4.75	3.030	7.439	0.001
Spanish Water Dog vs. Wheaten Terrier	7.20	3.707	13.962	0.001

Supplementary Table S6. Behavioural, environmental, and demographic variables derived from the owner-filled online canine behaviour questionnaire.

**Variable**	**Explanation**
Fear of dogs	Binomial (event/non-event) variable. Fearful dogs are reported to indicate fear of strange dogs often (40-100% of occasions) (event). Non-fearful dogs are not reported to indicate any fear of strange dogs nor have ever barked or growled to strange dogs (non-event).
Fear of strangers	Binomial (event/non-event) variable. Fearful dogs are reported to indicate fear of strangers often (40-100% of occasions) (event). Non-fearful dogs are not reported to indicate any fear of strangers nor have ever barked or growled to strangers (non-event).
Socialisation score	Continuous variable. A sum of the frequencies of how often (0 = never; 1 = 1-2 times during the puppyhood; 2 = 1-2 times during the puppyhood to 2 times per month; 3 = twice a month to twice a week; 4 = twice a week to once a day; 5 = several times a day) the dog met unfamiliar men, women and children, unfamiliar adult dogs, visited city or other place with traffic and many people, and travelled by car and bus when the dog was 7-16 weeks old. Minimum score 0, maximum score 35.
Urban environment score	Continuous variable. Describes the environmental land-use around the dog's home. The geographical coordinates for each home were derived from the addresses provided by the owners. The coverage of three land-use types (artificial surfaces, agricultural areas, forests and semi-natural areas) were calculated using CORINE2012 within a three-kilometre range around the homes. Through PCA, the coverages were simplified into one continuous variable, where higher scores indicate more urban environment.
Activities/training	Categorical variable. Describes how often the dog participates in activities or training. 1: never/seldom, 2: sometimes, 3: at least weekly.
Daily exercise	Categorical variable. Describes the amount of dog's daily exercise in hours. 1: < 1 hour, 2: 1-2 hours, 3: 2-3 hours, 4: > 3 hours.
Owner's dog experience	Binomial variable. Describes the owner's experience with dogs. 1: the dog is the owner's first dog, 2: the dog is not the owner's first dog.
Dogs in the family	Binomial variable. Describes the number of dogs in the family. 1: the dog is the only dog in the family, 2: there are other dogs in the family.
Family size	Categorical variable. Describes the size of the family where the dog lives. 1: single, 2: couple, 3: family with one or two adults and one child, 4: family with one or two adults and two children, 5: family with two or more adults and/or two or more children.
Weaning age	Categorical variable. The weaning ages were divided into four categories: 1: < 7 weeks of age, 2: at 7 weeks of age, 3: at 8 weeks of age, 4: > 8 weeks of age.
Daily time spent alone	Categorical variable. Describes the daily time that dog spends alone at home without the presence of people. 1: < 3 hours, 2: 3-6 hours, 3: 6-8 hours, 4: > 8 hours.
Body size	Categorical variable. Dogs were divided into three different body size categories, small, medium and large, based on the average height of the breed. Small, ≤ 35 cm; medium, 36-49 cm; large, ≥ 50 cm.

Supplementary Table S7. The AIC model selection and the final models in the logistic regression analyses. N(fear of dogs) = 5,343, N(fear of strangers) = 5,858.

Fear of dogs

Model	AIC	Body size added	Socialisation score added	Breed added	Sterilisation added	Urban environment score added	Activities/training added
Basic model (sex, age)	5237.3						
Body size	**5014.6**	5014.6					
Socialisation score	5159.5	**4932.4**					
Breed	5095.2	4943	**4859.8**				
Sterilisation	5198.5	4980.1	4898.6	**4822.5**			
Urban environment score	5197.2	5001	4898.2	4823.5	**4789.1**		
Activities/training	5164.3	4981	4904.1	4828.1	4796.9	**4767.6**	
Daily exercise	5187.7	4992.7	4919.8	4849.8	4813.9	4784.2	**4764.3**
Owner's dog experience	5229.7	5015.7	4930.7	4857.7	4821.9	4790.6	4769.3
Dogs in the family	5236.8	5016	4932.1	4856.7	4819.9	4790.2	4769.5
Family size	5243.8	5020.3	4937.2	4865.6	4827.5	4791	4770.4
Weaning age	5229.9	5016.2	4935.9	4863	4825.7	4792.5	4771.9
Daily time spent alone	5241.3	5018.6	4936.8	4864.5	4827	4793.2	4771.9
*The interaction term between sex and sterilisation was significant and improved the AIC value of the model, and thus the interaction term (sex*sterilisation) was included in the final model.*							
Sex*sterilisation							**4752.1**

Final model: sex, age, body size, socialisation score, breed, sterilisation, urban environment score, activities/training, daily exercise, sex*sterilisation.

Fear of strangers

Model	AIC	Socialisation score added	Breed added	Sterilisation added	Activities/ training added	Urban environment score added	Body size added	Weaning age added
Basic model (sex, age)	4960.5							
Socialisation score	**4833.2**	4833.2						
Breed	4862	**4733.8**						
Sterilisation	4923.9	4798.4	**4698.2**					
Activities/training	4920.8	4803.3	4708.5	**4677.9**				
Urban environment score	4957.9	4813.6	4718.7	4685.3	**4667.9**			
Body size	4926	4798.6	4722.6	4686	4670.4	**4661.5**		
Weaning age	4943.7	4822.8	4726.7	4690.8	4672.6	4663.1	**4657.4**	
Family size	4962.1	4832.7	4734.2	4697.2	4678.1	4664.6	4657.7	**4653.8**
Daily exercise	4953.1	4832.1	4735.7	4700.8	4681.2	4672.4	4666.1	4661.7
Daily time spent alone	4961.6	4831.9	4733.8	4697.5	4677	4667.2	4660.9	4656.9
Owner's dog experience	4960.2	4828.7	4731.2	4697.4	4678.2	4669.4	4663.2	4659.1
Dogs in the family	4960.2	4829.4	4728.5	4693.9	4677.1	4668.9	4662.4	4658.6
The interaction term between sex and sterilisation was tested but not included in the final model as it increased the AIC value of the model.								
Sex*sterilisation								4654.5

Final model: sex, age, socialisation score, breed, sterilisation, activities/training, urban environment score, body size, weaning age, family size.

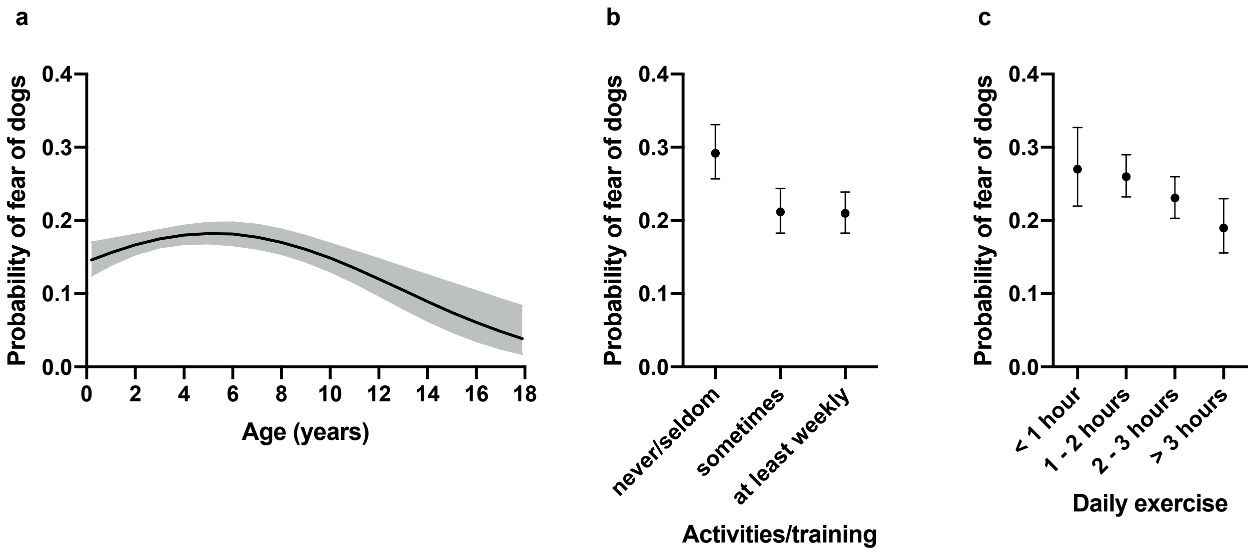

Supplementary Figure S1. The effects of age, activities/training, and daily exercise on fear of dogs in the logistic regression analysis. **(a)** Older dogs were less fearful as the likelihood of fear of dogs decreased after six years of age. **(b)** Dogs that participated in activities or training never or seldom were more afraid of dogs than dogs participating in activities sometimes or at least weekly. **(c)** Dogs getting less than one hour of daily exercise showed a higher likelihood of fear of dogs than dogs exercising more than three hours per day. In addition, dogs getting 1-2 hours of daily exercise showed a higher likelihood of fear of dogs than dogs exercising more than three hours per day. Grey lines (a) and error bars (b, c) indicate 95% confidence limits. N = 5,973.

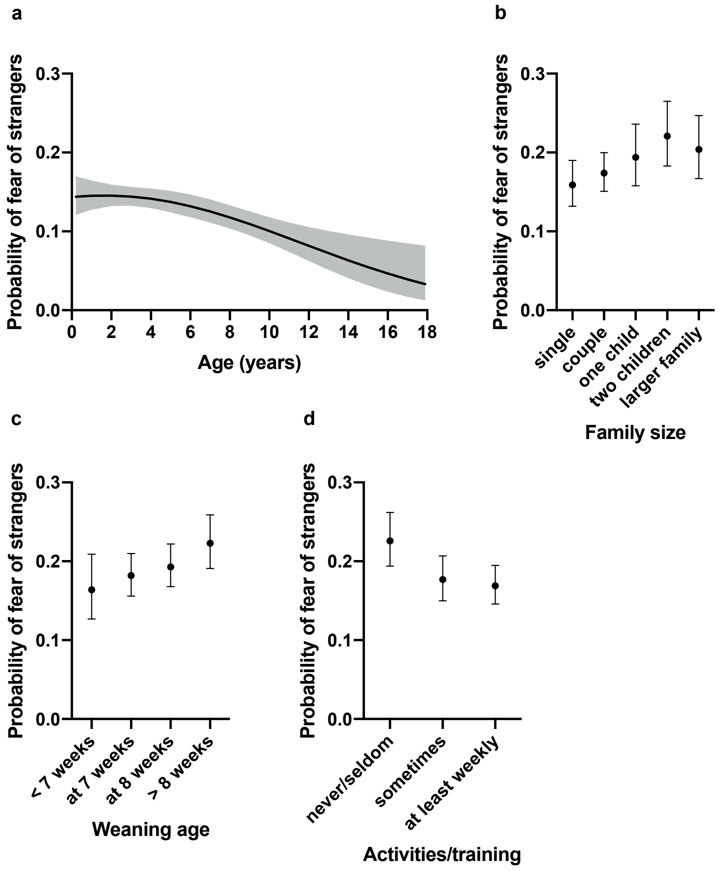

Supplementary Figure S2. The effects of age, family size, weaning age, and activities/training on fear of strangers in the logistic regression analysis. **(a)** Older dogs were less fearful as the likelihood of fear of strangers decreased after four years of age. However, the association between age and fear of strangers was not significant. **(b)** Dogs living with one ('single') or two adults ('couple') were less likely afraid of strangers than dogs living in families with one or two adults and two children ('two children'). **(c)** Dogs weaned later than eight weeks of age were more likely afraid of strangers than dogs weaned at seven weeks of age. **(d)** Dogs that participated in activities or training never or seldom were more afraid of strangers than dogs participating in activities sometimes or at least weekly. Grey lines (a) and error bars (b, c, d) indicate 95% confidence limits. N = 5,973.
